# Wilkerson's Dental Register and Examiner

**Published:** 1876-10

**Authors:** 


### BIBLIOGRAPHICAL.

*Dental Register and Examination Book*—By B. M. Wilkerson, D. D. S., M. D., Demonstrator of Operative Dentistry in the Baltimore College of Dental Surgery. Baltimore, 1876.

As a Dental Register this new work presents as complete ,convenient, and concise a system as it is possible to prepare ; and dental practitioners will find it as simple as it is useful.

The plan, according to which it is arranged, requires the least practicable amount of time or labor in registering dental operations and delineating the condition of the oral cavity, thus meeting the requirements of the operator in such a manner as to give the greatest satisfaction.

The diagrams, four in number on every page, are the special design of the author, and the most perfect for delineation which have yet been presented in a work of this kind. A book of this form containing 400 pages, will register 1600 patients, whereas the ordinary Case Book requires one entire page for each patient.

This new Register is also arranged for conveniently representing the character of professional services for each member of a family, without regard to numbers, with as much facility as the appointments are entered in a "pocket companion." Blanks for name, residence, &c., opposite each cut, prevent the possibility of confounding patients. This new work is gotten up in a handsome form with index, and printed on excellent paper.

